# The Potential of Polyethylene Terephthalate Glycol as Biomaterial for Bone Tissue Engineering

**DOI:** 10.3390/polym12123045

**Published:** 2020-12-18

**Authors:** Mohamed H. Hassan, Abdalla M. Omar, Evangelos Daskalakis, Yanhao Hou, Boyang Huang, Ilya Strashnov, Bruce D. Grieve, Paulo Bártolo

**Affiliations:** 1 Department of Mechanical, Aerospace and Civil Engineering, University of Manchester, Manchester M13 9PL, UK; abdalla.omar@manchester.ac.uk (A.M.O.); evangelos.daskalakis@manchester.ac.uk (E.D.); yanhao.hou@manchester.ac.uk (Y.H.); boyang.huang@manchester.ac.uk (B.H.); paulojorge.dasilvabartolo@manchester.ac.uk (P.B.); 2 Department of Chemistry, University of Manchester, Manchester M13 9PL, UK; Ilya.Strashnov@manchester.ac.uk; 3 Department of Electrical & Electronic Engineering, University of Manchester, Manchester M13 9PL, UK; bruce.grieve@manchester.ac.uk

**Keywords:** biomaterial, polyethylene terephthalate glycol, tissue engineering

## Abstract

The search for materials with improved mechanical and biological properties is a major challenge in tissue engineering. This paper investigates, for the first time, the use of Polyethylene Terephthalate Glycol (PETG), a glycol-modified class of Polyethylene Terephthalate (PET), as a potential material for the fabrication of bone scaffolds. PETG scaffolds with a 0/90 lay-dawn pattern and different pore sizes (300, 350 and 450 µm) were produced using a filament-based extrusion additive manufacturing system and mechanically and biologically characterized. The performance of PETG scaffolds with 300 µm of pore size was compared with polycaprolactone (PCL). Results show that PETG scaffolds present significantly higher mechanical properties than PCL scaffolds, providing a biomechanical environment that promotes high cell attachment and proliferation.

## 1. Introduction

Tissue engineering is an interdisciplinary field that applies the concepts of engineering and life sciences for the development of biological substitutes that maintain, restore or improve tissue or organ functions, and comprises two main strategies: scaffold-based and cell-laden approaches [1,2,3,4,5,6]. The scaffold-based approach, the most common strategy for bone tissue engineering applications, is based on the use of three-dimensional (3D) biocompatible and biodegradable porous structures that provide the substrate and the biomechanical environment for cells to attach, differentiate and proliferate [7,8,9,10,11,12]. Bone scaffolds must be produced using non-toxic materials (the materials must also degrade into non-toxic products), the degradation rate of the scaffold must be adjustable in order to match the rate of tissue regeneration, must present appropriate porosity, pore interconnectivity and pore structure (large number of pores may be able to enhance vascularization, while small pore sizes are preferable to provide large surface per volume ratio), should encourage the formation of the extra cellular matrix (ECM) by promoting cellular functions, should present sufficient strength and stiffness to withstand stresses in the host tissue environment and present adequate surface finish, guaranteeing that a good biomechanical coupling is achieved between the scaffold and the tissue [13,14,15,16,17].

Cell seeding on printed scaffolds depends on fast attachment of cell to scaffolds, high cell survival and uniform cell distribution, and these are strongly dependent on the scaffold material, architecture, surface stiffness and surface energy [2,18].

A variety of biodegradable materials have been used to produce the scaffolds, including a wide range of organic, inorganic and composite materials [15,19,20,21]. Among these materials, polycaprolactone (PCL), an aliphatic polyester, was extensively used by our group for bone tissue engineering applications. PCL scaffolds with different porosities and pore sizes were successfully used to support the attachment and proliferation of osteoblasts and mesenchymal stem cells and osteogenic differentiation [22,23,24,25]. In vivo studies, using PCL scaffolds to treat critical size calvaria defects in rats, also showed the ability to support new tissue formation [26]. However, PCL presents limited bioactivity, long degradation times and low mechanical properties not suitable for load-bearing applications [8]. In order to improve the bioactivity nature of the scaffolds, new surface modification strategies such as selective plasma modification, acetone vapor annealing surface treatment and dopamine grafting, and silica nanoparticles coating, were explored [22]. Bioactivity was also improved by mixing PCL with ceramic materials such as hydroxyapatite (HA) and tricalcium phosphate (TCP), which also allowed to increase mechanical properties and to accelerate the degradation process. PCL/HA/multi-wall carbon nanotubes, mimicking the structure of bone, were successfully produced using extrusion-based additive manufacturing [27]. Carbon nanomaterials such as graphene and carbon nanotubes were also used to improve mechanical properties and to allow the fabrication of electro-active scaffolds. In vivo studies showed that these scaffolds together with electrical stimulation significantly accelerate the formation of new bone and the production of more organized new bone [21]. However, in all these cases, as PCL is the main material, the overall biological and mechanical properties of the scaffolds were constrained by the individual PCL properties. Therefore, the rationale of this research is to identify a biocompatible and biodegradable synthetic polymer that can be used as an alternative to PCL, allowing to produce scaffolds with significantly better mechanical and biological properties. Polyethylene Terephthalate (PET) has been used in the medical field, for example for prosthetic vascular grafts, due to its good mechanical properties and biocompatibility [28]. Moreover, PET is also biodegradable, making it an interesting material for tissue engineering, but due to its high crystallinity, it presents significant printability limitations [29]. One route to overcome this printability limitation is through a glycol modification process, which allows to reduce the crystallinity level by disrupting the order of the polymer chain without compromising the biological characteristics of the initial polymer [24]. This strategy allows to obtain Polyethylene Terephthalate Glycol (PETG), investigated for the first time in this paper, as a potential material for tissue engineering applications. Thus, this research work addresses the following research questions: (1) is it possible to create PETG scaffolds, with significantly improved mechanical properties compared to PCL scaffolds, presenting compressive modulus in the range of trabecular bone? (2) Is it possible to create PETG scaffolds with superior biological performance compared to PCL scaffolds without any surface modification?

## 2. Materials and Methods

### 2.1. Materials

PETG, with a printing temperature between 195 and 220 °C, was purchased from RS components (Northants, UK). PCL pellets (Mw 50,000 Da, CAPA 6500) were supplied by Perstorp Caprolactones (Malmo, Sweden).

Matrix-assisted laser desorption/ionization time-of-flight mass spectrometry (MALDI-TOF MS) matrix was prepared using 2,5 Dihydroxybenzoic acid (DHB), Phenol and Chloroform (HPLC (High Performance Liquid Chromatography) grade), which were purchased from Sigma Aldrich (Dorset, UK).

Biological analysis was carried out using Human adipose-derived stem cells (hADSCs), MesenPRO RS^TM^ basal media, 2% (*v*/*v*) growth supplement, 1% (*v*/*v*) glutamine and 1% (*v*/*v*) penicillin/streptomycin, purchased from STEMPRO Invitroge, Thermo Fisher (Glasgow, UK) 2.2. Chemicals Analysis (MALDI-TOF MS).

PET is a common polymer used in many applications due to its high melting point, but it presents low printability. To overcome these problems, manufacturers such as RS components modified PET by adding Cyclohexanedimethanol (CHDM), producing PETG. MALDI-TOF MS was used to confirm the CHDM presence. This technique is based on the use of short, high-intensity laser pulses to form gas ions. In this case, the analyte molecules are not exposed to the laser but are added to a matrix of small organic molecules. The matrix shows strong absorption of the laser wavelength, improving efficiency and energy transfer [30]. Therefore, the matrix must have high electronic absorption for the used wavelength, stable under vacuum, low vapor pressure, good solubility in solvents that dissolve the analyte and good miscibility with the analyte in solid state.

The PETG sample was dissolved in phenol/chloroform 1/1 (*v*/*v*). 2,5 Dihydroxybenzoic acid (DHB) was used as a matrix. A dried droplet sample was prepared by putting 1 µL of a mixture of 1 mL of 2,5 DHB in Phenol/chloroform, 1/1 (*v*/*v*), as a matrix and 1 µL of polymer in phenol/chloroform, 1/1 (*v*/*v*), on the target plate [30,31]. In order to assess the chemical composition of PETG, the mass spectrometer Plus MALDI-TOF/TOFMS analyzer Axima Confidence (Shimadzu Biotech, Kiyamachi-Nijo, Japan) equipped with a Nitrogen laser emitting at 337.1 nm was used to achieve data acquisition in positive ion mode. Data acquisition and processing were carried out using the Kratos analyzer software (Kratos, Manchester, UK). The acceleration voltage was set to 20 KV and the extraction delay time used was 15,000 in reflector MS mode. All mass spectra were collected by averaging the signal of 1620 laser shots.

### 2.2. Scaffolds’ Fabrication

PETG and PCL scaffolds were produced using a filament-based additive manufacturing system (Flashforge Creator pro, Jinhua, China), considering a 0/90° lay-down pattern. PCL scaffolds were designed considering a pore size of 300 µm, and PETG scaffolds were designed considering three different pore sizes (PETG-300: 300 µm of pore size; PETG-350: 350 µm of pore size; PETG-450: 450 µm of pore size). Each scaffold was printed considering a total of 12 layers. The printing conditions are listed in Table 1.

### 2.3. Morphological Analysis

The scaffolds’ morphology was characterized using the Scanning Electron Microscopy (SEM) FEI ESEM Quanta 200 system (FEI Company, Hillsboro, OR, USA). The EMITECH K550X sputter coater (Quorum Technologies, Lewes, UK) was used for gold-coating the scaffolds prior to imaging. Imaging was carried out using 15 KV acceleration voltage. The obtained images were analyzed using the ImageJ software (Laboratory for Optical and Computational Instrumentation, University of Wisconsin, WI, USA), allowing to determine pore size, filament diameter and layer thickness. For each scaffold type, a total of nine measurements were considered.

### 2.4. Mechanical Compression Tests

Uniaxial compression tests were performed using an INSTRON X testing system (High Wycombe, UK) equipped with a 100 N load cell according to the ASTM D695-1. The scaffold samples were cut into 3 × 3 mm^2^ blocks. Samples (*n* = 6) were tested in a dry state at the displacement rate of 0.5 mm/min until the strain reached 0.3 mm/mm. The obtained strain-stress data were further processed and plotted using the software Origin (Origin Lab, Northampton, MA, USA).

### 2.5. Biological Analysis

#### 2.5.1. Cell Culture

Human adipose-derived stem cells (hADSCs) were used to investigate the cytotoxicity of the scaffolds. MesenPRO RSTM, Thermo Fisher (Glasgow, UK),1 basal media, 2% (*v*/*v*) growth supplement, 1% (*v*/*v*) glutamine and 1% (*v*/*v*) penicillin/streptomycin were used for cell culture. hADSCs (passage = 5) were cultured in an incubator (37 °C, 5% CO_2_ and 95% humidity) until an appropriate cell density (90%) was achieved before cell seeding. Scaffolds were sterilized in an 80% Ethanol solution for 4 h, followed by washing with Phosphate Buffered Saline (PBS) solution twice. The scaffolds were left to dry overnight in a sterile lamina flow cabinet. The sterilized scaffolds were transferred into 24-well plates and kept inside the incubator before the cell seeding. An 89 μL cell suspension containing 50,000 cells was added into each scaffold at day 0. The samples were then transferred to the incubator for 2 h, allowing hADSCs to attach to the scaffolds followed by adding 900 µL of cell culture media to cover the scaffold. The scaffolds were transferred into new 24-well plates on the following day and the cell culture media was replaced.

#### 2.5.2. Alamar Blue Assay

Cell metabolic activity was investigated using the Alamar Blue assay, Sigma Aldrich (Dorset, UK), which can be used as an indicator of cell proliferation as it quantifies the metabolic activity of cells. Tests were performed at days 1, 7 and 14 after cell seeding. At each time point, 90 µL of Alamar Blue solution was added to each scaffold and the scaffolds were incubated for 4 h. Then, 200 μL solution from each sample was transferred into a 96-well plate and tested using a microplate reader at 530 nm excitation and 590 nm emission. After the measurements, the scaffolds were washed three times with sterilized PBS to remove the residual Alamar Blue solution and new media was added. Cell culture media was changed every two days.

#### 2.5.3. Statistical Analysis

Statistical analysis was performed using one-way analysis of variance (ANOVA) with Tukey’s test. Differences were considered statistically significant at * *p* < 0.05, ** *p* < 0.01, *** *p* < 0.001 and **** *p* < 0.0001. GraphPad Prism software (GraphPad Software Inc., San Diego, CA, USA) was used in this research.

## 3. Results and Discussion

### 3.1. Chemical Analysis

**Figure 1:**
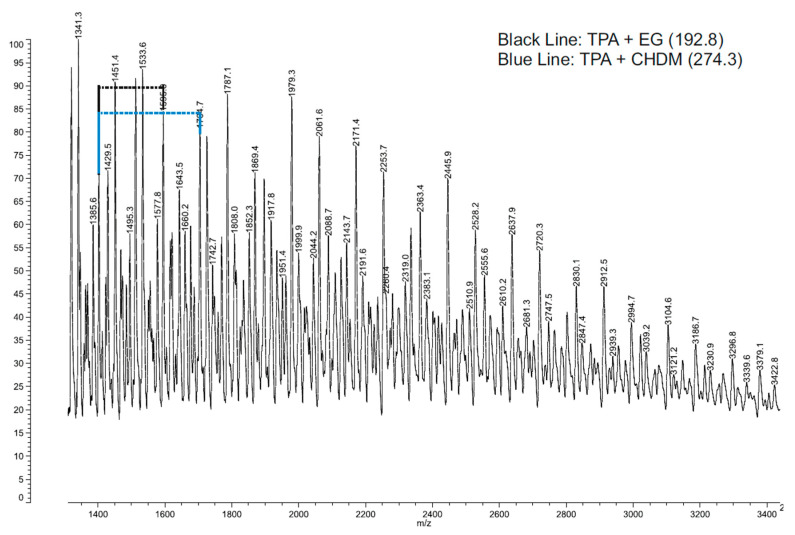
The Matrix-assisted laser desorption/ionization time-of-flight mass spectrometry (MALDI-TOF MS) spectrum of Polyethylene terephthalate glycol modified (PETG). Its characteristic repeated units are highlighted: 192.8 g/mol for the Terephthalic acid + Ethylene Glycol (TPA + EG) unit and 274.3 g/mol for Terephthalic acid + Cyclohexanedimethanol (TPA + CHDM).

The MALDI-TOF MS spectrum presented in Figure 1 shows a wide peak in the high mass range, and as a consequence, cannot provide structural information. Therefore, to identify the polymeric material under analysis, the repeating units is the only possible method [32,33]. In this case, the oligomers with low mass are considered to obtain the relevant structural information, such as repeated units and possible end groups [30,31]. In Figure 1, two lines were drawn to highlight the terephthalic acid (TPA) + ethylene glycol (EG) (black line), and TPA + CHDM (blue line). Besides the TPA + EG and TPA + CHDM peaks, the MALDI spectrum contains other peaks that correspond to fragment ions. The main constituent compounds and corresponding molecular weight values before the condensation reaction are presented in Table 2 [34].

It is also important to notice that the addition of CHDM to PET disturbs the structure of the polymer, thus reducing the crystallinity, melting temperature and mechanical properties, therefore increasing printability [35].

### 3.2. Scaffold Morphology

**Figure 2:**
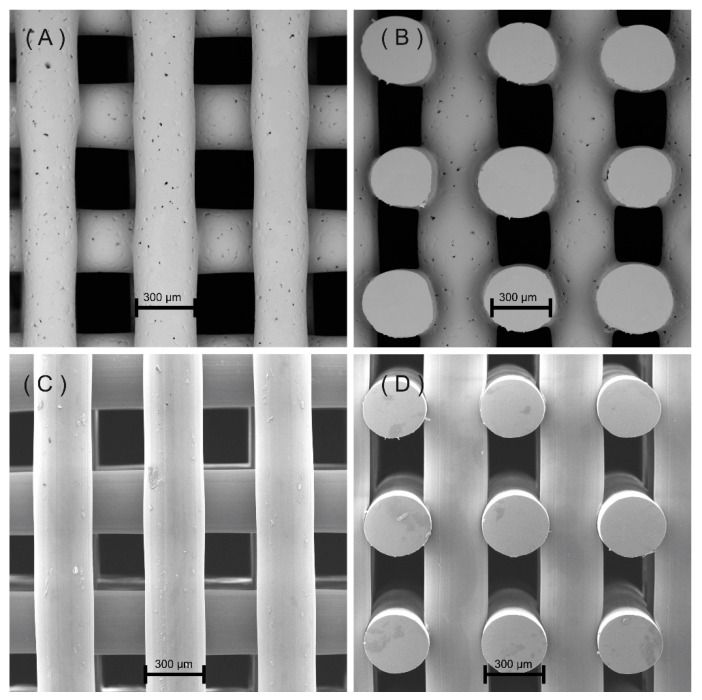
(**A**) Top view of a polycaprolactone (PCL) scaffold, (**B**) cross-section view of a PCL scaffold, (**C**) top view of a Polyethylene terephthalate glycol modified (PETG) scaffold, (**D**) cross-section view of a Polyethylene terephthalate glycol modified (PETG) scaffold. All scaffolds were designed considering a pore size of 350 µm.

Figure 2 shows the SEM micrographs of the printed scaffolds. As observed, scaffolds present well-defined geometries with regular distributed squared pores. The cross-section images (Figure 2B, D) show a uniform distribution of adjacent layers. Moreover, the presence of micro-pores on the surface of the PCL filaments can be observed from Figure 2A,C, which can be attributed to the material rheological characteristics and to the presence of brittle crystalline zones on the filament surface that collapse during the printing process [8]. This can also explain the better surface quality of PETG scaffolds as it is known that PETG is less crystalline than PCL [35]. However, further studies must be conducted to confirm this.

Contrary to PETG scaffolds, which present pore size (350 µm) and filament diameter (370 µm) values similar to the designed ones, a deviation was observed for PCL scaffolds (filament diameter of 340 ± 20, and pore size of 360 ± 10).

### 3.3. Mechanical Compression Test

**Figure 3:**
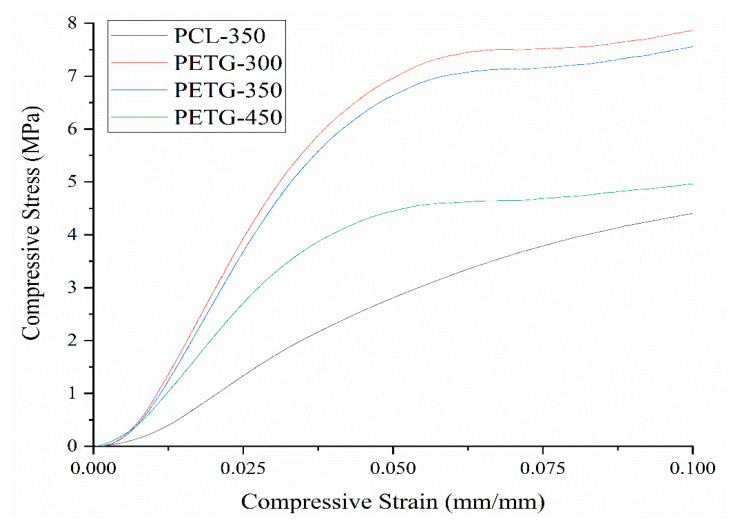
Stress vs. strain curves for PCL scaffolds and PETG scaffolds with different pore sizes.

**Figure 4:**
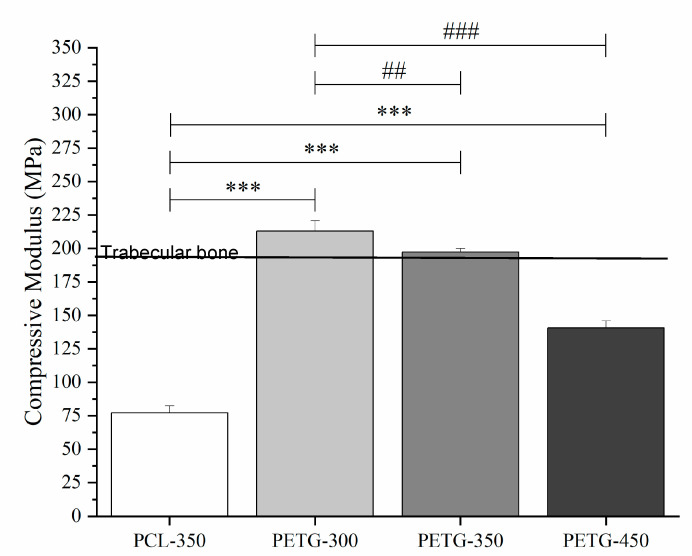
Compression modulus for the PCL scaffold and PETG scaffolds with different pore size. *** *p* < 0.001 compared with control (PCL), ^##^ *p* < 0.01 and ^###^ *p* < 0.001 compared with different pore size. The *** Statistical evidence (*p* < 0.001) is the one-way analysis of the mechanical compression test with the use of GraphPad Prism software and it is used to show the difference be-tween the results. The * is small difference and while more * are added the differences between the results are higher. * compared with PCL scaffolds, # compared with different pore size of PETG scaffolds.

**Figure 5:**
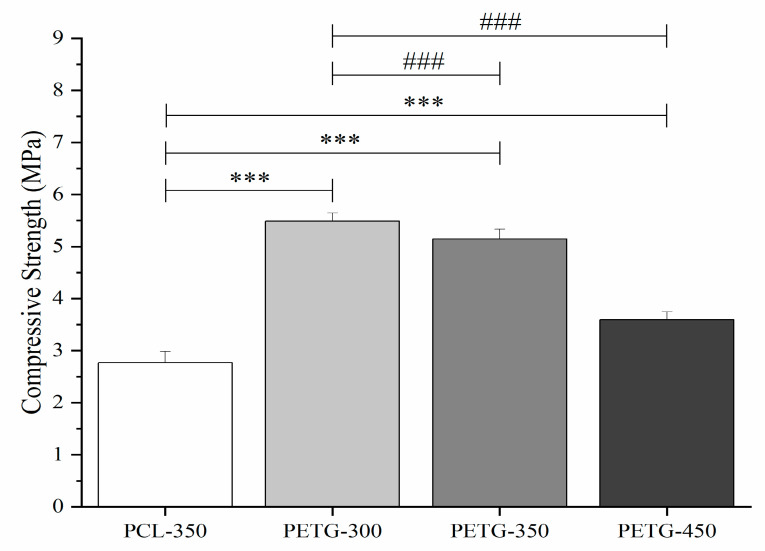
Compression strength values for the PCL scaffold and PETG scaffolds with different pore size. *** *p* < 0.001 compared with control (PCL), ^##^ *p* < 0.01 and ^###^ *p* < 0.001 compared with different pore size. The *** Statistical evidence (*p* < 0.001) is the one-way analysis of the mechanical compression test with the use of GraphPad Prism software and it is used to show the difference be-tween the results. The * is small difference and while more * are added the differences between the results are higher. * compared with PCL scaffolds, # compared with different pore size of PETG scaffolds.

Mechanical compression test results are presented in Figure 3, Figure 4 and Figure 5. Figure 3 presents the stress versus strain curves for all considered scaffolds, showing that, even for large pore sizes, PETG scaffolds exhibit higher mechanical properties. These curves were also used to calculate both compressive modulus and compressive strength. From Figure 4 and Figure 5, it is possible to observe that compared to PCL scaffolds, PETG scaffolds with the same pore size (350 µm) exhibit significantly higher compressive modulus and compressive strength, indicating that, from a mechanical perspective, and considering the same scaffold architecture, PETG scaffolds are more suitable for bone tissue engineering than PCL scaffolds. For PETG scaffolds, the results also show that by increasing the pore size from 300 to 450 µm, the compressive modulus of the PETG scaffolds decreases from 213 ± 12.2 to 141 ± 8.20 MPa. Results also show that by increasing the pore size, compressive strength decreases from 5.44 ± 0.19 to 3.55 ± 0.17 MPa. The average compressive modulus and strengths are presented in Table 3. Results also show that the PETG-300 and PETG-350 scaffolds have the same order of magnitude of mechanical properties as human trabecular bone (compressive modulus mean value of 194 MPa, and compressive strength mean value of 3.55 MPa) [36,37,38,39].

### 3.4. Biological Test

**Figure 6:**
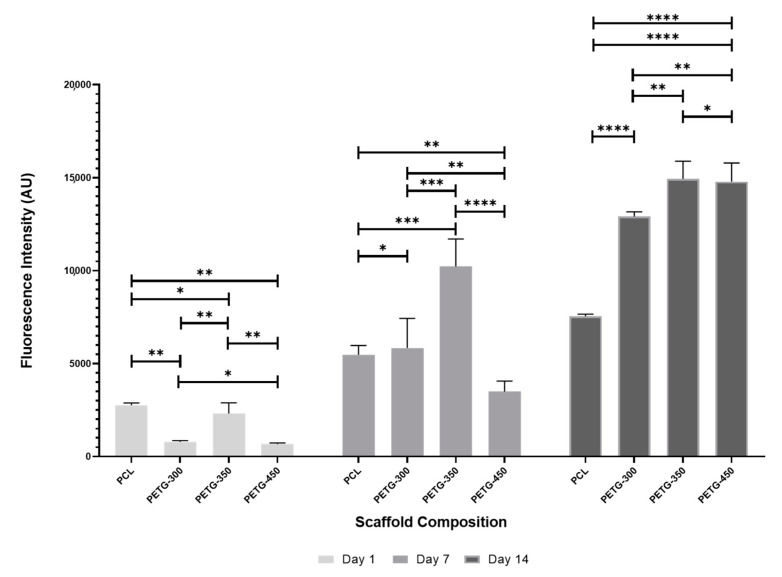
Alamar Blue results for both PCL and PETG scaffolds at days 1, 7 and 14 post-cell-seeding. * Statistical evidence (*p* < 0.05) analyzed by one-way analysis of variance (ANOVA) and Tukey’s post-test. The * Statistical evidence (*p* < 0.05), **, *** is the one-way analysis of variance (one-way ANOVA) and Tukey’s post hoc test with the use of GraphPad Prism software and it is used to show the difference be-tween the results. The * is small difference and while more * are added the differences between the results are higher.

Alamar Blue Assay was used to assess the metabolic activities of hADSCs seeded on PETG and PCL scaffolds at days 1, 7 and 14 of cell culture (Figure 6). Results show that the fluorescence intensities for all scaffolds increase by increasing the cell culture time, indicating that all scaffolds are cytocompatible and able to support cell attachment and spreading. At day 1, the fluorescence intensity of the PCL scaffold is statistically higher than PETG scaffolds. However, the fluorescence intensities of PETG-300 and PETG-350 scaffolds significantly increase and are statistically higher than those of PCL scaffolds at day 7 of cell culture. Moreover, the differences of the fluorescence intensities for all PETG scaffolds from day 1 to day 7 of cell culture are significantly higher than those of PCL scaffolds, suggesting a rapid colonization of hADSCs on PETG scaffolds. At day 14, all PETG scaffolds present statistically higher fluorescence intensities than PCL scaffolds. Although the differences of the fluorescence intensities from day 7 to day 14 decrease for all scaffolds, all PETG scaffolds present higher values than PCL scaffolds. These results show that PETG scaffolds present better cell affinity than PCL scaffolds. In terms of pore size, PETG-350 scaffolds show overall higher fluorescence intensities than other PETG scaffolds at days 1 and 7. However, PETG-450 scaffolds exhibit a fast increase on the cellular metabolic activity from day 7 to day 14 compared to other scaffolds. This might be attributed to low cell seeding efficiency at day 0. At day 14, both PETG-350 and PETG-450 scaffolds present similar fluorescence intensity results. Moreover, at day 14, these results seem to indicate higher cellular activity in scaffolds presenting larger pore sizes, which might be attributed to the fact that larger pore sizes allow better cell infiltration and reduce the risk of cell aggregation [40].

**Figure 7:**
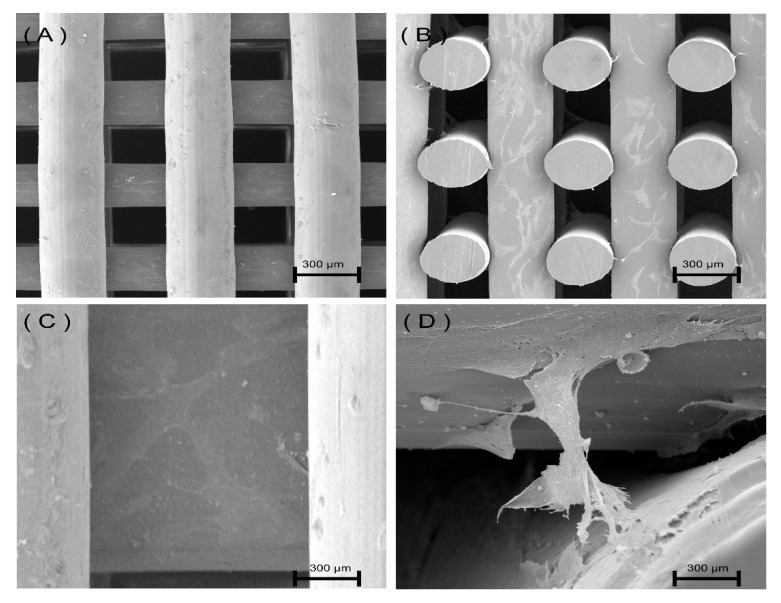
Cells on PETG-350 scaffolds after 14 days of cell seeding. (**A**) Top view image of the PETG scaffold, (**B**) cross-section image of PETG scaffold, (**C**) magnified image showing cells covering the PETG filament, (**D**) cells bridging adjacent layers.

SEM images of cells attached and spreading on PETG-350 scaffolds are shown in Figure 7. From this figure, it is possible to observe that cells are homogeneously distributed on the filaments, establishing cell–cell networks and bridging adjacent layers.

## 4. Conclusions

Scaffolds for bone tissue engineering should present appropriate mechanical properties and promote the right environment for cells to attach and proliferate. Among the different biocompatible materials being investigated for bone applications, PCL is the most common one. However, PCL scaffolds do not present suitable mechanical properties and these properties significantly decrease by increasing porosity or pore size, important parameters to allow vascularization, supplying oxygen and nutrients to cells inside the scaffold. This paper investigated, for the first time, the potential use of PETG as an alternative material to PCL. This material, obtained by adding CHDM to PET (as confirmed by MALDI analysis), was successfully printed, and the produced scaffolds exhibited a well-defined geometry. Results also showed that PETG scaffolds present higher compressive modulus and compressive strength than PCL scaffolds, not only for the reference case (350 µm of pore size) but also when we considered PETG scaffolds with larger pore sizes (450 µm). Moreover, PETG scaffolds also showed better biological behavior, as observed from the Alamar Blue assay. In this case, it was also possible to observe high cellular activity in scaffolds presenting large pore sizes. Considering both mechanical and biological performances, results seem to suggest that the PETG scaffold with pore size of 350 µm is, among the different scaffolds considered in this study, the most appropriate one.

## Figures and Tables

**Table 1:** Scaffold printing parameters.

Parameters	Values
Layer thickness (mm)	0.33
Filament diameter (mm)	0.35
Nozzle temperature (°C)	230
Bed temperature (°C)	60
Printing speed (mm/s)	20

**Table 2:** Chemical constituents for PETG.

Compound	Molecular Formula	Molecular Weight (g/mol)
Terephthalic acid (TPA)	C_8_H_6_O_4_	166.13
Ethylene Glycol (EG)	(CH_2_OH_2_)	62.07
Cyclohexanedimethanol (CHDM)	C_8_H_16_O_2_	144.21

**Table 3:** Compressive mechanical properties.

	PCL	PETG-300	PETG-350	PETG-450
Compressive Modulus (MPa)	76.1 ± 7.53	213± 12.2	196 ± 6.53	141 ± 8.20
Compressive Strength (MPa)	2.56 ± 0.26	5.44 ± 0.19	5.09 ± 0.21	3.55 ± 0.17
